# Gene expression differences consistent with water loss reduction underlie desiccation tolerance of natural *Drosophila* populations

**DOI:** 10.1186/s12915-023-01530-4

**Published:** 2023-02-16

**Authors:** Vivien Horváth, Sara Guirao-Rico, Judit Salces-Ortiz, Gabriel E. Rech, Llewellyn Green, Eugenio Aprea, Mirco Rodeghiero, Gianfranco Anfora, Josefa González

**Affiliations:** 1 Institute of Evolutionary Biology, CSIC, UPF, Barcelona, Spain; 2 Agriculture Food Environment Centre (C3A), University of Trento, San Michele All’adige (TN), Italy; 3 Research and Innovation Centre, Fondazione Edmund Mach, San Michele All’adige (TN), Italy

**Keywords:** Cuticular hydrocarbons, Water content, Respiration rate, Post-transcriptional regulation, tRFs, Insect physiology

## Abstract

**Background:**

Climate change is one of the main factors shaping the distribution and biodiversity of organisms, among others by greatly altering water availability, thus exposing species and ecosystems to harsh desiccation conditions. However, most of the studies so far have focused on the effects of increased temperature. Integrating transcriptomics and physiology is key to advancing our knowledge on how species cope with desiccation stress, and these studies are still best accomplished in model organisms.

**Results:**

Here, we characterized the natural variation of European *D. melanogaster* populations across climate zones and found that strains from arid regions were similar or more tolerant to desiccation compared with strains from temperate regions. Tolerant and sensitive strains differed not only in their transcriptomic response to stress but also in their basal expression levels. We further showed that gene expression changes in tolerant strains correlated with their physiological response to desiccation stress and with their cuticular hydrocarbon composition, and functionally validated three of the candidate genes identified. Transposable elements, which are known to influence stress response across organisms, were not found to be enriched nearby differentially expressed genes. Finally, we identified several tRNA-derived small RNA fragments that differentially targeted genes in response to desiccation stress.

**Conclusions:**

Overall, our results showed that basal gene expression differences across individuals should be analyzed if we are to understand the genetic basis of differential stress survival. Moreover, tRNA-derived small RNA fragments appear to be relevant across stress responses and allow for the identification of stress-response genes not detected at the transcriptional level.

**Supplementary Information:**

The online version contains supplementary material available at 10.1186/s12915-023-01530-4.

## Background

Global climate changes, such as increased temperature and unpredictable changes in precipitation, pose a severe and widespread impact on organisms, from human health and food security, to species distribution and biodiversity [1–4]. Of the natural disasters caused by climate change, droughts are among the costliest [5]. These unpredictable patterns of precipitation are causing an increase in aridity and the expansion of drylands in many regions [6]. Water related challenges are threatening many species, but insects are particularly vulnerable due to their small size and thus large surface area to volume ratio [7–9]. In recent studies, a substantial (47–80%) decline in the abundance of some insect species was reported, partly due to climate change [10, 11]. Because insects represent most of the animal diversity, and include many economically and ecologically extremely relevant species, such as bees, mosquitoes, and moths, understanding the adaptive responses of insects to climate change is crucial [12, 13].

Most of the insect-related climate change studies have so far focused on the effect of increased temperature [14–19], while response to desiccation conditions caused by changes in rainfall, humidity, and water availability have received less attention [12, 18, 20]. *Drosophila* species are good models to study the physiological and genetic basis of adaptation to dry environments, as species of this genus have adapted to diverse climatic conditions during their recent evolutionary history, including arid regions [21–24]. Indeed, geographical variation for desiccation tolerance among populations of several *Drosophila* species has been found [25–35]. Among these species, *D. melanogaster* is an ideal model organism to further analyze desiccation stress response given its worldwide geographic distribution, the wealth of functional knowledge, and the genetic tools available [36].

There are several genome-wide studies investigating the underlying genetic architecture of desiccation tolerance in *D. melanogaster* [32, 37–40]. However, knowledge on the genome-wide transcriptomic response is still limited, as most studies focused on the analysis of a few candidate genes in laboratory selected lines [41–46]. Transcriptomic analysis are relevant as they allow to identify changes in gene expression that can be due to genetic and epigenetic variation, as well as informing on the biological processes affected by the studied condition. The few transcriptomic studies available to date suggest that stress sensing, stress response, immunity, signaling, and gene expression pathways are relevant for desiccation tolerance [38, 39, 44].

While the role of single-nucleotide polymorphisms (SNPs) and chromosomal inversions in gene expression changes in response to desiccation have been investigated [38, 39], the potential role of transposable elements (TEs) as gene regulators in this stress response has not yet been studied. TEs are very powerful mutagens that can affect gene expression through a variety of molecular mechanisms [47, 48]. Indeed, the adaptive role of TE insertions in response to stress conditions has been reported across organisms [49–51]. Similarly, despite the growing evidence that points to tRNA-derived small RNA fragments (tRFs) as relevant post transcriptional gene regulators in stress response, their role in desiccation stress has not yet been studied either [52–55].

Finally, integrating physiology into the analysis of the genomic basis of desiccation stress response should help us better understand the underpinnings of this ecologically relevant trait. While three main physiological mechanisms have been related to desiccation tolerance in insects—water loss reduction, increased bulk water content, and water loss tolerance—the latter appears to be a less common mechanism in *Drosophila* [18, 56–59]. Reduced water loss rate appears to be the most common mechanism to survive desiccation [22, 57, 59–62]. Water loss happens mostly by two routes; the first occurs through the spiracles during the open phase in respiration [59, 63]. The second is related to the cuticular hydrocarbons hindering transpiration (CHCs), which are the most prominent fatty acid-derived lipids on the insect body surface [58, 64]. The variation in water loss through the cuticle has been related to the amount, chain length, and saturation of CHCs, with the most notable being a negative correlation between the length of the hydrocarbon chain and rates of water loss [8, 58, 60, 65]. Finally, the role of increased bulk water content in desiccation tolerance is still not clear. While flies more tolerant to desiccation stress were found to have higher bulk water content [8, 35, 62, 66, 67], in other studies either no significant differences were described [68] or higher water content was associated with lower desiccation tolerance [69]. To date, the physiological response to desiccation has been mainly studied in xeric *Drosophila* species or in *D. melanogaster* strains selected for desiccation tolerance. As such, a comprehensive picture in natural *D. melanogaster* populations is still not available [32–35, 70].

**Fig. 1:**
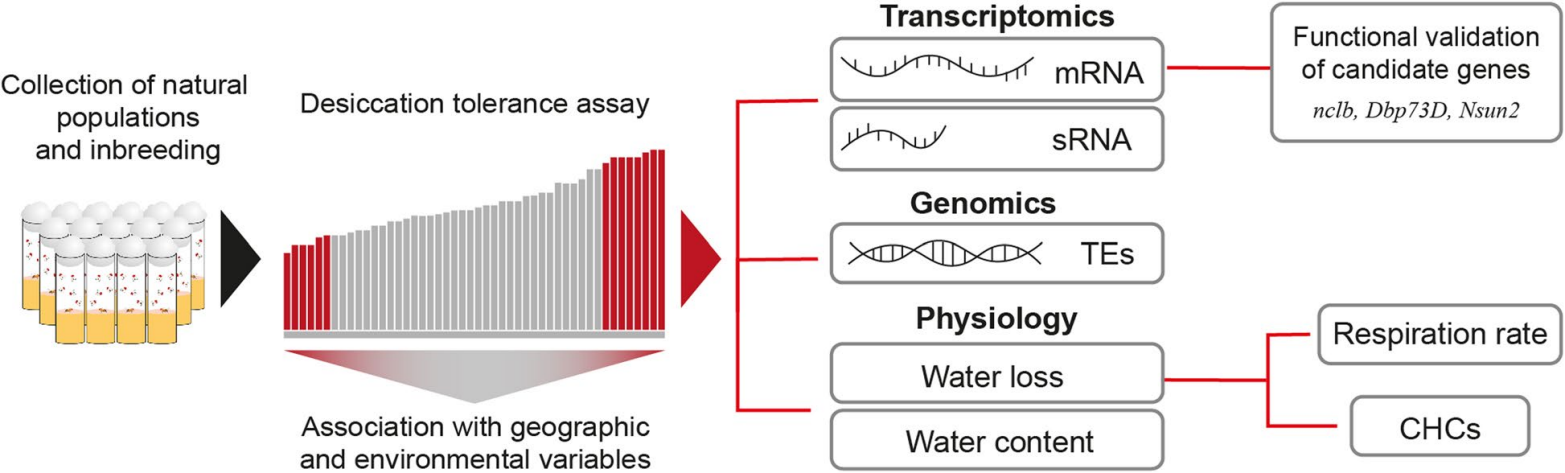
Schematic representation of the analyses performed in this work. Flies were collected from nine European populations and inbred for up to 21 generations. Desiccation tolerance was measured for all the wild-derived strains and association with geographic and environmental variables was performed to investigate the variation in desiccation tolerance across strains. Transcriptomics, genomics, and physiological assays were performed with the subset of strains from the extremes of the phenotypic distribution. Three of the genes identified in the differential gene expression analysis were functionally validated. To further analyze the differences in water loss identified, both respiration rate assays and characterization of the cuticular hydrocarbon composition was performed

In this work, we assessed the natural variation for desiccation tolerance in European *D. melanogaster* strains and analyzed whether geographical and environmental variables were associated with the variation identified. We then integrate transcriptomics, including sequencing of mRNAs and small RNAs, genomics, physiological assays, including water content, water loss, and respiration rate, and cuticular hydrocarbons analysis to further understand the differential response to desiccation tolerance of natural *D. melanogaster* strains (Fig. 1).

## Results

### Altitude and evaporation correlate with desiccation tolerance in European natural *D. melanogaster* strains

**Fig. 2:**
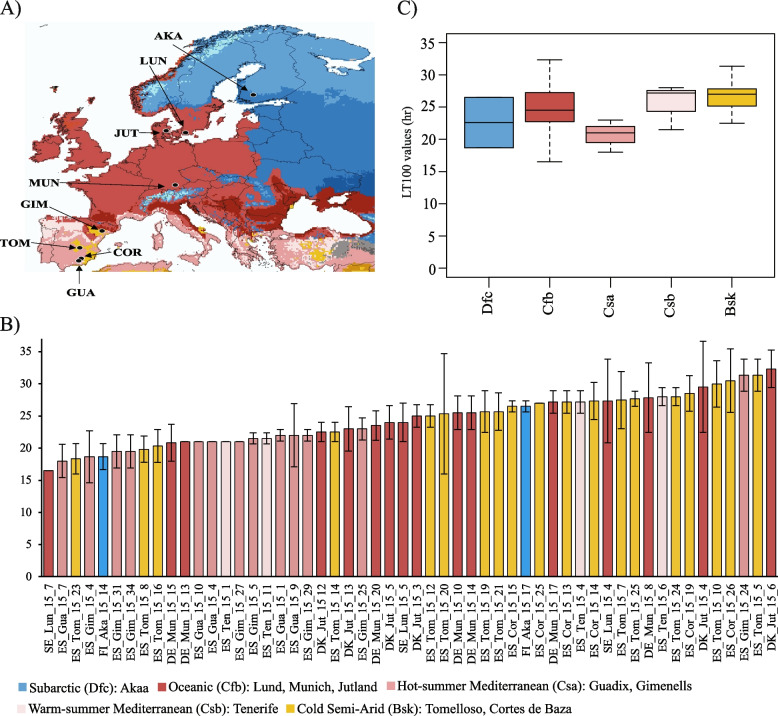
Natural variation in desiccation survival across European natural *D. melanogaster* populations. **A** Geographical origin of the nine populations analyzed in this study. The location of the populations is indicated with arrows in a map of Europe colored based on the Köppen-Geiger climate zones, except for Tenerife, which is not shown in the map (Additional file 1. Table S1). **B** Desiccation survival of European natural populations. LT_100_ values for the 59 inbred strains (three replicates/strain) that showed less than 10% control mortality are shown (Additional file 3. Table S2A). The *Y*-axis represents the average hour when the flies in all the replicates were dead, and the *X*-axis represents the individual strains colored by the climate zone in which they were collected. Data are presented as mean values ± SD. **C** Boxplot of the distribution of the LT_100_ values of the strains, grouped by climate zones (Additional file 3. Table S2A). The boxplot shows the median (the horizontal line in the box), 1st and 3rd quartiles (lower and upper bounds of box, respectively), and minimum and maximum (lower and upper whiskers, respectively)

To measure the variability in desiccation tolerance in *D. melanogaster* natural populations, we exposed females from 74 inbred strains collected in nine European locations to low humidity conditions (< 20% humidity) (Fig. 2A and Additional file 1: Table S1). These nine populations belong to five different climate zones including cold (subarctic), temperate (oceanic, hot-summer Mediterranean, and warm-summer Mediterranean climates), and arid (cold semi-arid) climates. LT_50_ and LT_100_ values of the European strains were significantly correlated (Spearman’s rho = 0.928, *p*-value < 0.01). LT_50_ values of individual strains from these nine populations, representing the time when half the flies were dead, ranged from 12.5 to 29.7 h, which is wider than those found in North American strains (17.9 to 20 h [32]; Additional file 2: Fig. S1A and Additional file 3: Table S2A). LT_100_ values, representing the time when all the flies were dead, varied between 16.5 and 32.3 h (Fig. 2B and Additional file 3: Table S2A). Similar differences in desiccation tolerance were found in populations from India collected from different latitudes, where the more tolerant strains survived about twice as long as the sensitive ones (LT_100_ = 28.6 vs. 13.6) [34], while in Australian populations, the LT_100_ variation was smaller (14.2 to 17.5 h) [41]. Thus, European populations showed similar or wider ranges of variation in survival times to desiccation stress compared with strains from other continents.

Flies from temperate climates have been shown to be more tolerant to desiccation stress than flies from tropical climates [32, 41, 57, 71]. We thus tested whether there were significant differences in survival among flies from the five climates in our dataset (Fig. 2). While no differences were found in the average LT_50_ values, we found significant differences in the average LT_100_ values (ANOVA, *p*-value = 0.467 and 0.023 for LT_50_ and LT_100_, respectively; Fig. 2C and Additional file 3. Table S2A). Pairwise comparisons showed significant differences between LT_100_ values of strains from the cold semi-arid (*Bsk*) and hot-summer Mediterranean (*Csa)* climate zones: the cold semi-arid strains were more tolerant (Tukey comparison, *p*-adj = 0.01; Fig. 2C and Additional file 3: Table S2A).

Finally, desiccation tolerance has been correlated with altitude, latitude, and environmental variables such as annual precipitation and minimum temperature [20, 32, 34, 72]. We did not find a significant correlation between latitude, longitude, or altitude and the desiccation tolerance of the strains (LT_100_; linear model, *p*-value = 0.648 for altitude, *p*-value = 0.853 for latitude, and *p*-value = 0.686 for longitude; Additional file 3: Table S2A). On the other hand, we found that the LT_100_ values significantly correlated with the interaction of altitude and evaporation (*p*-value = 0.0005, adjusted *R*-squared: 0.135; Additional file 3: Table S2A; see Methods).

### Desiccation tolerant and sensitive strains differed in the number, the direction of expression change, and the function of genes that respond to stress

To investigate the transcriptional response to desiccation stress, we generated whole female RNA-seq data for three tolerant and three sensitive strains chosen from the extremes of the LT_50_ distribution (Additional file 2: Fig. S1B; see Methods) [73]. We used *Transcriptogramer*, which takes into account protein–protein interactions to perform differential expression of functionally associated genes (clusters) [74], to investigate the overall response to desiccation stress, by analyzing the six strains together (“All DEGs”). We also investigated whether tolerant (“Tolerant DEGs”) and sensitive (“Sensitive DEGs”) strains differed in their transcriptomic response to desiccation stress.

**Fig. 3:**
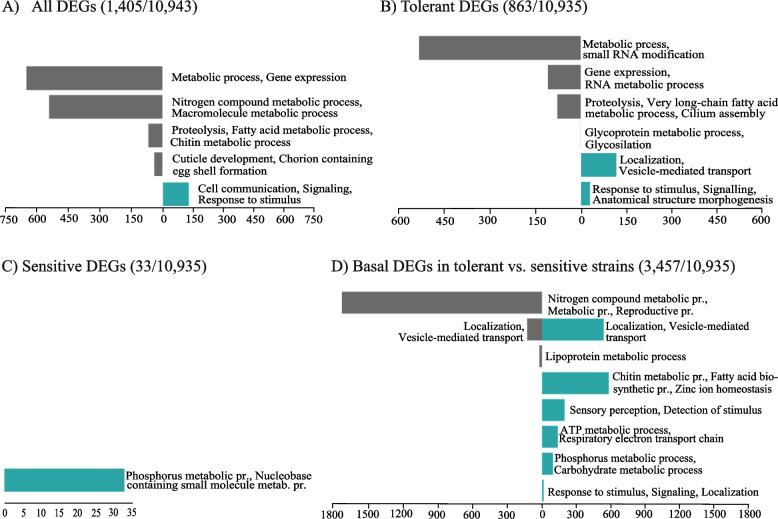
Biological process GO enrichment of the differentially expressed gene clusters identified by *Transcriptogramer*. **A** GO enrichment of the DEGs when all six strains (three tolerant and three sensitive) were analyzed together (Additional file 4: Table S3A). **B** GO enrichment of the DEGs in tolerant strains (Additional file 4: Table S3B). **C** GO enrichment of the DEGs in sensitive strains (Additional file 4: Table S3C). **D** GO enrichment of the DEGs in basal conditions when comparing tolerant vs sensitive strains (Additional file 4: Table S3D). In all the plots, the *X*-axis represents the number of genes contained in the clusters. Gray indicates enrichment for downregulated genes, while blue indicates enrichment for the upregulated genes. In parenthesis, the number of DEGs/number of genes analyzed are given. For each condition, three replicates of three tolerant and three replicates of three sensitive strains were sequenced

When analyzing the six strains together, we identified five clusters of DEGs with 92% (1292 out of 1405) of the genes downregulated (Fig. 3A and Additional file 4: Table S3A) [73]. Genes in the two clusters with the biggest number of downregulated genes were enriched for metabolic processes (RNA, nitrogen compounds, macromolecules) and gene expression functions (Fig. 3A and Additional file 4: Table S3A). In the other two downregulated clusters, genes were enriched for chitin metabolic process, fatty acid biosynthetic process, and cuticle development (Fig. 3A and Additional file 4: Table S3A). Note that genes related to gene expression, RNA metabolism, and chitin metabolism have been previously associated with desiccation stress response [28, 38, 39]. On the other hand, upregulated genes were enriched for cell communication, signaling, and response to stimulus functions (Fig. 3A and Additional file 4: Table S3A). Response to stimulus and environmental sensing have also been previously associated to desiccation stress response in *D. melanogaster* and *D. mojavensis* [28, 39].

The same pattern was found in the “Tolerant DEGs” with 83% of the genes downregulated (716 out of 863 Fig. 3B and Additional file 4: Table S3B) [73]. This result is not unexpected, as 86% of the “Tolerant DEGs” overlap with the “All DEGs.” The four downregulated and two upregulated clusters were enriched for similar GO terms as the ones found in the “All DEGs” analysis, with the upregulated clusters also enriched for localization and transport functions (Fig. 3B and Additional file 4: Table S3B).

On the other hand, a very small number of genes (33 genes) were differentially expressed in sensitive strains, and they were all upregulated and enriched for nucleotide metabolic and catabolic processes (Fig. 3C and Additional file 4: Table S3C) [73]. This low number of genes suggests that sensitive strains have a limited coordinated desiccation stress response.

We found a considerable overlap among our DEGs, considering “All DEGs,” “Tolerant DEGs” and “Sensitive DEGs,” and genes previously related to desiccation stress response (379 out of 1524 DEGs (25%); Additional file 5: Table S4A), including four DEGs that affect the cuticular composition of *D. melanogaster* [75], and 10 DEGs that overlap with the core set of candidate genes identified in the cross-study comparison carried out by Telonis-Scott et al. (2016) (Additional file 5: Table S4B) [39]. While we found overlap with previously described candidates, our transcriptomic analysis also identified new candidate genes (see below).

Overall, tolerant and sensitive strains differed in their transcriptomic response to stress not only in the number of desiccation-responsive genes but also in the direction of the change of expression and in the gene functions (Fig. 3A–C).

### Tolerant strains have a higher level of basal expression of desiccation-responsive genes

Basal transcriptional states have been associated with differential response to cold stress and bacterial infection [62, 76]. We thus compared the gene expression in tolerant versus sensitive strains in basal conditions. We found eight gene clusters containing 3457 DEGs, which included 851 genes that have been previously related to desiccation stress, eight of them affecting the cuticular composition of *D. melanogaster* (Additional file 4: Table S3D, Additional file 5: Table S4A) [73]. Moreover, 15 of the DEGs in basal conditions overlap with the core set of candidate genes identified in the cross-study comparison of Telonis-Scott et al. (2016) (Additional file 5: Table S4B) [39].

Downregulated gene clusters were mostly enriched in metabolic processes, while upregulated clusters contained genes related to response to stimulus, ion transport, sensory perception, cell communication, metabolic processes such as chitin metabolic processes, fatty acid elongation, and very long chain fatty acid biosynthetic process (Fig. 3D and Additional file 4: Table S3D).

Overall, our results showed that tolerant and sensitive strains differed in their basal gene expression levels, with tolerant strains showing higher levels of basal expression of genes previously associated with desiccation tolerance such as response to stimulus, chitin metabolic process, and fatty acid elongation (Fig. 3D and Additional file 4: Table S3D).

### Desiccation-responsive genes are enriched for highly expressed genes in the ovary

We tested whether DEGs in response to stress, and DEGs when comparing tolerant and sensitive strains in basal conditions were enriched for highly expressed genes in the ovary, as has been previously described [37]. Using the *Drosophila Gene Expression Tool* (DGET) [77], we found that stress-response DEGs, including hub DEGs (see Methods), were enriched for highly expressed genes in the ovary (hypergeometric test, *p*-value < 0.0001, for all comparisons; Additional file 6: Table S5A-C, Additional file 7. Table S6A-C). On the other hand, basal DEGs were enriched for highly expressed genes in head, digestive system, carcass, and ovary (hypergeometric test, *p*-value < 0.0001, for all comparisons). However, basal hub DEGs were enriched for highly expressed genes in the ovary and digestive system (hypergeometric test, *p*-value < 0.0001, for all comparisons, Additional file 6: Table S5D, Additional file 7: Table S6D).

### *nclb *and *Dbp73D* genes affect desiccation tolerance in *D. melanogaster*

Besides detecting genes previously known to play a role in desiccation tolerance, our transcriptomic analysis also identified new candidate genes (Additional file 4: Table S3). We chose three hub genes among the ones with the highest maximal clique centrality (MCC) values, *nclb*,* Nsun2*, and *Dbp73D*, to perform functional validation experiments (Additional file 7: Table S6; see Methods; [78]). These genes were (i) mostly expressed in the ovary and digestive system, (ii) related to gene expression and RNA methylation (Table 1), and (iii) were downregulated in the “All DEGs” and “Tolerant DEGs” groups (Additional file 4: Table S3).

**Table 1:** Functional validation of three candidate desiccation-responsive genes. Expression level and survival of gene disruption and RNAi reciprocal crosses relative to the control strains (F = female and M = male). At least three replicates were analyzed in all cases (Additional file 8: Table S7C)

Gene name/Flybase ID	Gene function	Gene disruption/RNAi (stock number)	qRT-PCR results (one-tailed *t*-test *p*-value)	Survival of the gene disruption/RNAi strain compared with background strain	Log-rank test (*p*-value)
*nclb*/ FBgn0263510	Regulation of gene expression	*P-element* insertion in first intron (#21,138)	Lower expression (0.00190)	Higher survival	< 0.0001
		RNAi (#41826) F x Gal4-6c M	Lower expression (0.0139)	Higher survival	< 0.0001
		RNAi (#41826) M x Gal4-6c F	Lower expression (0.03.39)	Higher survival	< 0.0001
*Nsun2*/ FBgn0026079	RNA methylation, tRNA methylation	*P-element* insertion in first intron (#33452)	Lower expression (0.01.19)	Lower survival	< 0.0001
		RNAi (#62495) F x Gal4 M	Lower expression (0.0003)	Higher survival	< 0.0001
		RNAi (#62495) M x Gal4 F	Lower expression (0.0134)	Higher survival	< 0.0001
*Dbp73D*/ FBgn0004556	RNA binding	RNAi (#36131) F x Gal80ts M	Lower expression (0.00374)	Lower survival	< 0.0001
		RNAi (#36131) M x Gal80ts F	Lower expression (0.00461)	Lower survival	< 0.0001

**Fig. 4:**
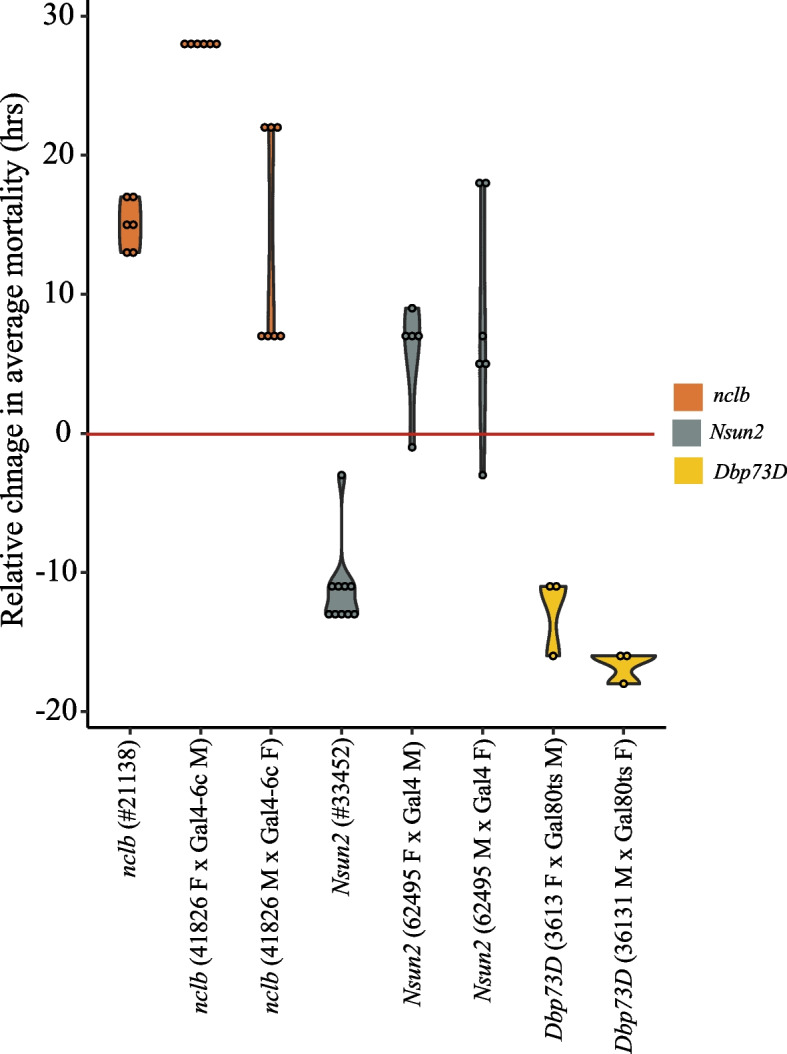
Functional validation of *nclb*, *Nsun2*, and *Dbp73D* genes. Relative change in average mortality at the end of the desiccation assay comparing gene disruption and knock-down (RNAi) strains for the three candidate desiccation-responsive genes with control strains. At least three replicates were analyzed in all cases. Additional file 8C: TableS7C

In the case of *nclb*, the insertional mutant and the two reciprocal crosses of the RNAi transgenic line analyzed showed a lower level of expression and higher survival in desiccation stress conditions when compared with background strains (Log-rank test, *p*-value < 0.0001 in all three comparisons; Fig. 4, Table 1, and Additional file 8: Table S7). These results are consistent with the observed downregulation of the *nclb* gene in tolerant strains in desiccation stress conditions (Additional file 4: Table S3).

For *Nsun2*, we found that while the expression of the gene in the insertional mutant and the two reciprocal crosses of the RNAi transgenic line was lower, the survival to desiccation stress was lower for the insertional mutant but higher for the RNAi reciprocal crosses (Log-rank test, *p*-value: < 0.0001 for the three comparisons; Fig. 4, Table 1 and Additional file 8: Table S7). These results are consistent with a role of *Nsun2* in desiccation tolerance and suggest that the effect of this gene is background dependent.

Finally, for *Dbp73D*, the two reciprocal crosses performed with the RNAi line showed lower gene expression and lower survival under desiccation stress conditions (Log-rank test, *p*-value: < 0.0001; Fig. 4, Table 1, and Additional file 8: Table S7). This result suggests that the effect of *Dbp73D* on desiccation tolerance is also background dependent as we found this gene to be downregulated in tolerant strains.

Overall, we found that all three genes affect the desiccation survival of the flies, however in some cases this effect depends on the genetic background.

### Differentially expressed genes in response to desiccation are not enriched for TE insertions

TEs have previously been shown to affect gene expression in response to stress in *D. melanogaster* (e.g. [79, 80]. As a first step towards the analysis of the role of TE insertions in desiccation stress response, we tested whether DEGs in response to desiccation stress and DEGs when comparing tolerant vs. sensitive strains basal expression were enriched for TE insertions. We used the de novo TE annotations for the six genomes analyzed to identify TEs located either inside genes or in the 1 kb upstream and downstream gene regions [81, 82]. When taking into account all DEGs in response to desiccation stress, we found that TEs were depleted nearby DEGs compared with the distribution of TEs nearby genes in the genome (Table 2 and Additional file 9: Table S8). Similar results were obtained for basal DEGs, where TEs were found to be depleted compared with the distribution of TEs nearby genes genome-wide (Table 2 and Additional file 9: Table S8). Although DEGs were not significantly enriched for nearby TE insertions, we cannot discard that some of the identified TEs located nearby DEGs could be responsible for the differences in expression detected. Additional functional validation experiments would be needed to test this hypothesis.

**Table 2:** Number of differentially expressed genes located nearby transposable element (TE) insertions. d.f.: degrees of freedom (Additional file 9: Table S8A-D)

	Upregulated genes with TEs nearby	Chi-square test (post hoc test *p* value)	Downregulated genes with TEs nearby	Chi-square test (post hoc test *p* value)	Total number of DEGs with TEs nearby	Chi-square test
All DEGs	11 (9.7%)	1.1543, df = 1, *p*-value = 0.2826	127 (9.8%)	23.249, df = 1, *p*-value = 1.423e − 06, (1.22E − 04, depleted)	138 (9.8%)	18.895, df = 1, *p*-value = 1.381e − 05 (4.60E − 05, depleted)
Tolerant DEGs	19 (13%)	1.4124, df = 1, *p*-value = 0.2347	54 (7.5%)	3.7558, df = 1, *p*-value = 0.05262	73 (8.5%)	1.469, df = 1, *p*-value = 0.2255
Sensitive DEGs	1 (3%)	NA	0	NA	1 (3%)	NA
Basal DEGs	226 (14.3%)	4.7876, df = 1, *p*-value = 0.02866 (depleted)	189 (10%)	3.7778, df = 1, *p*-value = 0.05194	415 (12%)	10.144, df = 1, *p*-value = 0.001448 (5.23E − 03, depleted)

### tRNA-derived fragments differentially target genes in response to desiccation stress

While tRNA-derived small RNAs (tRFs) are known to inhibit the translation of protein coding genes during starvation stress and aging, due to complementary sequence matching, their potential role in desiccation stress response has not yet been assessed [52, 53]. To test whether tRFs could play a role in desiccation stress response, we sequenced small RNAs using whole females under control and desiccation stress conditions in the tolerant and sensitive strains. We identified the genes targeted by tRFs in tolerant and sensitive strains and focused on those genes that were differentially targeted in response to desiccation stress, i.e., genes that gain or lose targeting (Additional file 10: Table S9; see Methods) [83]. For tolerant strains, 106 genes were targeted by tRFs in response to stress while 332 genes lost targeting in response to stress (Additional file 10: Table S9A). While the same number of genes were targeted by tRFs in response to stress in sensitive strains, the number of genes that lost targeting was smaller (53 genes; Additional file 10: Table S9A). Most genes that lost targeting in response to stress overlap between sensitive and tolerant strains (87%: 46 out of 53). This overlap is much smaller for the genes that are targeted in response to stress suggesting that tRFs target different genes in tolerant and sensitive strains (37%: 39 out of 106).

We tested whether DEGs in tolerant and sensitive strains overlapped with genes that were post-transcriptionally targeted by tRFs (Additional file 10: Table S9B). The overlap ranged from 1 to 11% suggesting that different sets of genes are controlled at the transcriptional and post-transcriptional levels. The overlap at the biological process level was also small, suggesting that transcriptional and post-transcriptional control of desiccation responsive genes is different both at the gene and at the biological process level (Fig. 3, Table 3, and Additional file 10: Table S9C). We finally tested whether the genes that were differentially targeted by tRFs in response to desiccation stress in tolerant and sensitive strains have been previously identified as desiccation-responsive genes (Additional file 10: Table S9D). Up to 5% (40/423) of genes targeted by tRFs have previously been identified as desiccation-responsive genes (Additional file 10: Table S9D).

**Table 3:** GO enrichment analysis using DAVID tool of the genes that gain or lose targeting by tRFs in response to desiccation stress in tolerant and in sensitive strains (Additional file 10: Table S9C)

	**Targeting appears in response to desiccation stress**	**Enrichment score**	**Targeting disappears in response to desiccation stress**	**Enrichment score**
**Tolerant strains**	Response to stimulus	1.7	Nervous system development	3.9
			Regulation of developmental process, neurogenesis	2.8
			Regulation of metabolic process	2.4
			Response to stimulus	2
			Regulation of signaling	1.9
**Sensitive strains**	Nervous system development	1.9	NS	NA
	Signaling, response to stimulus	1.4		
	Cell adhesion	1.4		
	Response to growth factor	1.3		

Overall, these results suggest that tRFs do play a role in desiccation stress response, with the majority of tRFs targeting different genes in tolerant and sensitive strains, and allow the identification of new candidate desiccation-responsive genes.

### Differences in the cuticular hydrocarbons and in respiration rate are associated with reduced water loss in desiccation tolerant strains

Besides investigating the transcriptional response to stress and the potential role of TEs and tRFs in this response, we also characterized the physiological response to desiccation in tolerant and sensitive strains. We measured three physiological traits that have been associated with the level of desiccation tolerance in flies—water content, rate of water loss and respiration rate—and we characterized the cuticular hydrocarbon composition of tolerant and sensitive strains [34, 35, 60, 69].

#### Tolerant strains have lower bulk water content

**Fig. 5:**
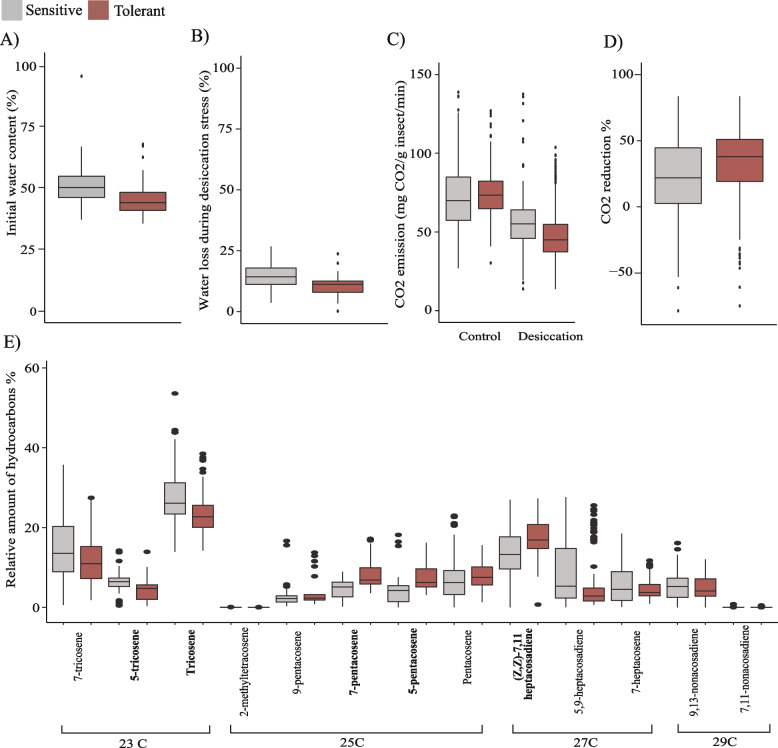
Desiccation-related physiological traits and cuticular hydrocarbon variation in natural European populations. **A** Initial water content in sensitive and tolerant strains (10 tolerant and 10 sensitive strains, 10 replicates/strain; Additional file 11: Table S10A). **B** Percentage of water loss during desiccation stress in sensitive and tolerant strains (10 tolerant and 10 sensitive strains, 4–5 replicates/strain, except one strain with 3 and one strain with 2 replicates; Additional file 11: Table S10B). **C** Respiration rate under control and desiccation stress conditions in sensitive and tolerant strains (three tolerant and three sensitive strains, 3 replicates/strain; Additional file 12: Table S11B-G). **D** Percentage of CO_2_ decrease (respiration) in response to desiccation stress in sensitive and tolerant strains (three tolerant and three sensitive strains, 3 replicates/strain; Additional file 12: Table S11B-G). **E** Relative amount of cuticular hydrocarbons in sensitive (gray) and tolerant (red) strains. Hydrocarbons that showed significant differences between sensitive and tolerant strains are depicted in bold (10 tolerant and 10 sensitive strains, 7–10 replicates except for one strain with 4 replicates; Additional file 13: Table S12). All boxplots show the median (the horizontal line in the box), 1st and 3rd quartiles (lower and upper bounds of box, respectively), and minimum and maximum (lower and upper whiskers, respectively)

We first checked the bulk water content in females from the 10 most tolerant and 10 most sensitive strains of the LT_50_ distribution (Additional file 2: Fig. S1A; see Methods). We found that the tolerant strains had significantly lower initial water content compared to the sensitive ones (45% *vs.* 50% on average, Wilcoxon test, *p*-value: < 0.0001, Fig. 5A and Additional file 11: Table S10A), which is consistent with previous studies [69].

#### Tolerant strains lose less water during desiccation stress compared with sensitive strains

Next, we quantified the amount of water loss by measuring the female flies’ weight before and after three hours of desiccation stress. We found that on average sensitive strains lose 15% of their water content while the tolerant strains lose 10% (Wilcoxon test, *p*-value < 0.0001; Fig. 5B and Additional file 11: Table S10B). These results are consistent with previous studies performed both in populations selected for desiccation stress tolerance and natural populations [34, 62].

#### Tolerant strains decrease their respiration rate more in desiccation stress conditions

We measured the respiration rate of tolerant and sensitive strains in control and desiccation stress conditions. We compared GLMM with and without interaction between the experimental condition (control and desiccation stress) and the phenotype of the strains (tolerant and sensitive), and we found that the model including the interaction fitted the data better (LRT, *p*-value < 0.0001) (Additional file 12: Table S11). Tolerant strains have a higher respiration rate in control conditions compared with sensitive strains (Fig. 5C). However, after desiccation stress, the sensitive strains lower their respiration rate by 23% on average while the tolerant strains do so by 35% (Fig. 5D).

#### Tolerant strains have higher relative amount of desaturated hydrocarbons

The level of cuticular transpiration, which is another influential factor in water loss, depends on the composition of the cuticle. Thus, we next analyzed the cuticular hydrocarbon (CHC) composition in tolerant and sensitive strains in control conditions (see Methods). Overall, we identified 13 main hydrocarbons with chain lengths varying between 23 and 29 carbons, including three saturated (n-alkanes) and ten desaturated compounds (alkene, alkadiene) (Fig. 5E, Table 4, Additional file 13: Table S12). We performed principal component analysis (PCA) to explore the variability of the strains in terms of CHC composition. We found that although tolerant and sensitive strains differed in the relative amounts of individual hydrocarbons (see below), neither PC1 nor PC2 separated the 10 tolerant from the 10 sensitive strains when considering the total CHC composition (Additional file 2: Fig. S2A). However, PC1 did separate the three tolerant from the three sensitive strains for which we characterized the transcriptomic response to desiccation stress and explained 63.72% of the variation (Additional file 2: Fig. S2B). Note that these six strains are on the extremes of the phenotypic distribution for water loss measurements (Additional file 11: Table S10B).

**Table 4:** Cuticular hydrocarbons (CHCs) identified in the 10 most tolerant and 10 most sensitive strains. CHCs with a statistically significant different amount between the tolerant and sensitive strains are marked in bold. Linear retention index denotes the behavior of compounds on a gas chromatograph according to a uniform scale determined by the internal standard [84]. The data presented in this table is based on 10 tolerant and 10 sensitive strains and 7–10 replicates/strain (except for one strain with 4 replicates) (Additional file 13: Table S12)

Linear retention index	Component	Formula (acronym)	Hydrocarbon type	Saturated/Desaturated
2279	7-Tricosene	C23H46 (7-C23:1)	Alkene	Desaturated
2290	**5-Tricosene**	C23H46 (5-C23:1)	Alkene	Desaturated
2298	**Tricosane**	C23H48 (n-C23)	Alkane	Saturated
2464	2-Methyltetracosane	C25H52 (2-Me-C25)	Alkane	Saturated
2473	9-Pentacosene	C25H50 (9-C25:1)	Alkene	Desaturated
2480	**7-Pentacosene**	C25H50 (7-C25:1)	Alkene	Desaturated
2480	**5-Pentacosene**	C25H50 (5-C25:1)	Alkene	Desaturated
2498	Pentacosane	C25H52 (n-C25)	Alkane	Saturated
2657	**(Z,Z)-7,11 Heptacosadiene**	C27H52 (7,11-C27:2)	Alkadiene	Desaturated
2670	5,9-Heptacosadiene	C27H52 (5,9-C27:2)	Alkadiene	Desaturated
2693	7-Heptacosene	C27H54 (7-C27:1)	Alkene	Desaturated
2841	9,13-Nonacosadiene	C29H56 (9,13-C29:2)	Alkadiene	Desaturated
2872	(Z,Z)-7,11-Nonacosadiene	C29H56 (7,11-C29:2)	Alkadiene	Desaturated

Tolerant strains had a higher relative amount of desaturated hydrocarbons (Wilcoxon test, *p*-value = 0.004) and a higher desaturated to saturated balance ratio compared with sensitive strains, as previously reported (Wilcoxon test, *p*-value = 0.004191; Additional file 13: Table S12; [69]. However, the percentage of 7,11:Cn alkadienes, which was previously reported to be negatively correlated with desiccation tolerance, was found to be slightly positively correlated in our strains (Spearman’s correlation = 0.203, *p*-value = 0.02 [70]. The relative percentage of longer chain hydrocarbons (≥ 27C) was not correlated with desiccation tolerance (Spearman’s correlation =  − 0.19, *p*-value = 0.828; Additional file 13. Table S12) [70]. However, in Foley and Telonis-Scott (2011) hydrocarbons with ≥ 27 carbons represented approximately half of the total CHC composition, while in our European strains, compounds with ≥ 25 carbons or more are the most abundant. This is likely explained by the different latitudes in which populations from these two studies were collected (33). If we focus on ≥ 25C compounds, we did find a higher percentage in the tolerant compared with sensitive strains (Wilcoxon test, *p*-value < 0.0001). Moreover, the percentage of ≥ 25C compounds positively correlated with the LT_100_ (Spearman’s correlation = 0.178, *p*-value = 0.041) (Additional file 13: Table S12). In line with these results, we also found that tolerant strains had a higher relative percentage of 5-pentacosene, 7-pentacosene, and 7,11-heptacosadiene, which are all compounds with ≥ 25C (Wilcoxon test, *p*-value < 0.0001 in all cases; Fig. 4D), while sensitive strains had a higher relative percentage of 5-tricosene and tricosene (Wilcoxon test, *p*-value: < 0.0001 in all cases), which are compounds with 23C (Fig. 5E).

Overall, tolerant and sensitive strains at the extremes of the phenotypic distribution differed in the global CHC composition, and all the strains tested differed in the relative percentage of individual CHCs. While we did not find some of the previously described correlations between particular CHC and desiccation tolerance, this is most likely explained by the different CHC composition of the European strains, in which CHC with 25 or more carbons (instead of CHC with 27 or more carbons) represent approximately half of the total CHCs.

## Discussion

In this study, we combined several experimental approaches, including genomics, transcriptomics and physiological assays, to further understand the genetic basis of desiccation tolerance (Fig. 1). We first characterized the variation in desiccation tolerance of natural European *D. melanogaster* strains from cold, temperate, and arid regions (Fig. 2). While in previous studies in Australia [41, 57], South America [71], and North America [32], temperate strains were found to be more tolerant compared to tropical ones, here we showed that in Europe, strains from arid regions are similar or more tolerant to desiccation compared to strains from temperate regions (Fig. 2C). While tropical climates are mainly characterized by the temperature of the coldest month (*T*_cold_ ≥ 18), temperate climates are also characterized by the temperature of the hottest month (*T*_hot_ > 10 and 0 < *T*_cold_ < 18). On the other hand, arid climates are defined by the mean annual precipitation (MAP < 10 × *P*_threshold,_ where the value of *P*_threshold_ depends on which season the majority of the precipitation occurs [85]. We also found that variation in desiccation resistance in European strains can be partly explained by altitude and evaporation. The importance of altitude was previously shown in Indian populations of *D. melanogaster*, where flies from highlands were more desiccation tolerant compared with flies from lowlands [34]. Besides geographical and environmental variables, variation in recombination characteristics, i.e. crossover rate and crossover interference, has also been reported to be associated with differences in desiccation tolerance across strains [86].

Besides characterizing the natural variation in desiccation stress tolerance, we sought to uncover the physiological traits that influence this variation and the coordinated response of genes which orchestrate it. In control conditions, the tolerant strains showed a higher level of respiration rate (Fig. 5D and Additional file 12: Table S11). Consistent with this result, genes related to respiration, *i.e.*, respiratory electron transport chain, and cellular respiration, were upregulated in tolerant strains in control conditions (Fig. 3D and Additional file 4: Table S3D). Genes related to ion transport were also upregulated in the tolerant strains compared to the sensitive ones in control conditions (Additional file 4: Table S3D). Ion homeostasis genes have been suggested to be involved in water retention by the Malpighian tubules and other cells throughout the body [32, 38] and have been related to desiccation survival before [87].

Although tolerant strains have a higher respiration rate in control conditions, following desiccation stress, they lower their respiration rate more than the sensitive ones (Fig. 5D) and were consistently found to lose less water (Fig. 5B). Reduced water loss after desiccation stress has previously been found in desiccation tolerant *D. melanogaster* strains and xeric *Drosophila* species [8, 22, 34, 35, 62, 68]. Moreover, we found that genes related to metabolic processes were downregulated after desiccation stress in the tolerant strains, thus probably causing a lowered metabolism (Fig. 3B). Reduction in the metabolic rate is known to reduce the need to open the spiracles, which is consistent with tolerant flies losing less water [88–90].

We found that besides differences in respiration rate, differences in cuticular hydrocarbon (CHC) composition between tolerant and sensitive strains are also likely to contribute to reduced water loss in tolerant strains (Fig. 5E). Moreover, genes related to very long chain fatty acid elongation (bigger than 20C [91]) and chitin metabolic process were upregulated in the tolerant strains under basal conditions (Fig. 3D). However, they were downregulated after desiccation stress (Fig. 3B). This result suggests that the tolerant strains do not improve the water retaining properties of the cuticle during desiccation stress by increasing the production of cuticular proteins as has been suggested in *D. mojavensis*, where genes involved in chitin metabolism and cuticle constituents were found to be upregulated after desiccation stress [28]. Thus, it seems that the tolerant strains have a different, more favorable CHC composition under basal conditions compared with sensitive strains, which might be related with their capacity to better survive low humidity conditions.

Tolerant strains also showed an increased expression of genes related to response to stimulus, signaling, localization, and transport after desiccation stress (Fig. 3B). Furthermore, tolerant strains also upregulated genes related to sensory perception and detection of chemical stimulus under basal conditions compared to sensitive strains (Fig. 3D). These results suggested that the response to desiccation has an important environmental sensing component, as has been previously suggested in *D. melanogaster* and in *D. mojavensis* [28, 38, 39]. Indeed *Pkd2* (*Polycystic kidney disease 2*), *pyx* (*pyrexia*), and *pain* (*painless*) genes involved in hygroreception, a sense that allows the flies to detect changing levels of moisture in the air, were found to be differentially expressed in tolerant strains under basal conditions [38, 39]. Moreover, 27% (14 out of 52) of odorant binding proteins (*Obp*) that are also associated with hygroreception [46], were upregulated in tolerant strains in basal conditions, which also underpins the importance of environmental sensing in control conditions (Additional file 4: Table S3D).

The transcriptomics characterization of desiccation tolerant and sensitive strains also allowed us to pinpoint new desiccation-responsive gene candidates. Two of the three desiccation-responsive genes validated in this work, *Dbp73D* and *Nsun2*, have already been shown to be important in stress responses in *Arabidopsis thaliana* and mice [92, 93]. In *Arabidopsis thaliana*, *Dbp73D* mutants show increased tolerance to salt, osmotic stress and heat stress [93]. In mice, the *Nsun2* gene has been shown to be repressed amid oxidative stress, and the loss of *Nsun2* gene altered the tRF profiles in response to oxidative stress [92]. In Drosophila, *Nsun2* mutants have been shown to cause loss of methylation at tRNA sites [94]. These results suggest that *Dbp73D* and *Nsun2* appear to be involved in general stress response across organisms and that tRFs are part of the stress response. In this work, we also investigated the potential role of tRFs in desiccation stress response and found tRFs that differentially target genes in tolerant and sensitive strains in response to desiccation stress (Table 3). Previous works in *D. melanogaster* pinpoint the role of tRFs in cellular starvation response through the regulation of translation of both specific and general mRNAs [53]. We found that the overlap between DEGs and genes targeted by tRFs was small suggesting that different genes are controlled at the transcriptional and post-transcriptional levels. However, further functional validation analysis is needed to confirm the role in desiccation stress response of the genes targeted by tRFs.

## Conclusions

Overall, we found that *D. melanogaster* natural populations from arid regions are similar or more tolerant to desiccation stress compared with populations from temperate regions and that this tolerance correlates with altitude and evaporation. Combining gene expression profiling with physiological trait analysis allowed us to pinpoint the genetic basis of the physiological response to desiccation stress. We found that differences in gene expression levels in basal conditions were more prevalent than differences in gene expression in response to desiccation stress, suggesting that basal gene expression levels should be incorporated into the analysis of differential stress survival. Both differences in basal gene expression and in stress response were consistent with differences in the cuticular hydrocarbon composition and in the respiration rate, which altogether contribute to explain the water loss reduction of tolerant strains. Transcriptomic analyses also allowed us to identify new candidate desiccation-responsive genes, three of which were functionally validated. Finally, we identified genes that are differentially targeted by tRFs in response to stress, suggesting that tRFs previously involved in starvation resistance and aging could also play a role in desiccation stress response in *D. melanogaster*. The results obtained in this work can also have considerable practical implications, for example by helping to understand the potential for spreading, adaptation, and damage of economically important species in the light of ongoing climate change. An example might be the case of an invasive alien species belonging to the same genus, *Drosophila suzukii*, one of the major agricultural pests worldwide [95, 96].

## Methods

### Fly husbandry

Flies were collected in 2015 from nine European locations by members of the *DrosEU* consortium (Fig. 2 and Additional file 1: Table S1). These nine locations belong to five different climate zones according to the Köppen-Geiger climate classification: subarctic (*Dfc*), oceanic (*Cfb*), hot-summer Mediterranean (*Csa*), warm-summer Mediterranean (*Csb*), and cold semi-arid (*Bsk*) (Additional file 1: Table 1; [50, 97]. In total, 74 inbred strains were generated from the aforementioned natural populations. Flies were inbred for 20 generations, except for two strains which were inbred for 21 generations and 11 strains that were too weak to continue with the inbreeding process and were thus stopped before reaching 20 generations (Additional file 1: Table S1). Fly stocks were kept in vials at 25 °C and 65% relative humidity with 12 h day and night cycles.

### Desiccation survival experiments

For each of the 74 strains analyzed, three replicates of 15 individuals each (except for 63 replicas with 10–18 individuals) were performed for desiccation stress conditions and three replicates (except for two strains with 1–2 replicates) of 15 individuals each (except for 4 replicates with 9–10 flies) were performed for control conditions (Additional file 3: Table S2A). In both conditions, 4–8 day-old females were used. Also in both conditions, the vials were closed with cotton and sealed with parafilm to stop the airflow. In treated conditions, flies were put in empty vials and three grams of silica gel (Merck) were placed between the cotton and the parafilm, so they were starved and desiccated. Control vials were prepared similarly, except that they contained 1 mL of 1% agar on the bottom of the vial to prevent desiccation (agar provides hydration but is not a food source; [43]. Temperature and humidity were continuously monitored using three *iButton*s (Mouser electronics) (Additional file 3: Table S2). Fly survival was monitored every 4 h until hour 12 and at shorter intervals afterwards (1 to 3.5 h). Flies that died before the first survival check were considered to have been injured during the experiment setup and were not included in the analysis (Additional file 3: Table S2).

For 15 out of the 74 strains, > 10% mortality was observed in control conditions and thus were not further analyzed (Additional file 3: Table S2A). Lethal time 50 (LT50, the time when 50% of the flies are dead) was calculated using *Probit* analysis [98] for all the strains (59 strains). LT100 was calculated for all the strains for which mortality data were collected until the end of the experiment (54 strains). Data are presented as mean values ± SD.

Since our LT50 and LT100 data is measured in intervals and we have the same number of observations, we first confirmed that the data was monotonic. We then determine the relationship between LT50 and LT100 values by performing a one-tailed Spearman’s rank order correlation test using the SPSS software (v. 29.0.0.0).

### *Correlation of LT*_*100*_* with environmental and geographical variables*

To test if the LT_100_ data followed a normal distribution, we used the Shapiro–Wilk normality test. As the data were normally distributed, we performed an ANOVA analysis to test if there were differences in the average of the LT_100_ values among different climate zones (anova (lm (LT100 ~ Climate, data = lt100)), followed by a Tukey’s post hoc test to check which climates differ from each other (TukeyHSD (aov (lm (LT100 ~ Climate, data = lt100)))). We then tested if the LT_100_ was correlated with geographical or environmental variables. The geographical variables used were longitude, latitude, and altitude (Additional file 1: Table S1). For environmental data, we used two different sources: (i) *WorldClim* (www.worldclim.org; [99] and (ii) Copernicus (*ERA5*) (Additional file 1: Table S1) [100]. Environmental variables related to temperature and precipitation have been shown to explain the greatest variability in desiccation resistance in Drosophila [20]; so, we used the 19 bioclimatic variables from WorldClim, which were derived from the monthly temperature and rainfall values between 1970 and 2000 and the yearly maximum and minimum temperature. We used the R package *raster* (v. 2.6–7) for downloading these data [101]. We also used evaporation (the accumulated amount of water that has evaporated from the Earth’ surface, including a simplified representation of transpiration (from vegetation), into vapor in the air above) and solar radiation (clear-sky direct solar radiation at surface) data from the year previous to the collection date obtained from ERA5 database from Copernicus [102]. Evaporation is known to cause desiccation stress, and solar radiation has been shown to affect mortality and development under desiccation stress in gastropods [103, 104].

Multicollinearity is very common when working with geographical/environmental variables, so we calculated the variance inflation factor (VIF) for the geographical/environmental variables used in this study in order to remove variables that correlate with each other [105]. Environmental and geographical variables were sequentially removed based on the highest VIF, until the VIF number was lower than five. A linear regression analysis, with the three variables with VIF < 5 (altitude, longitude and evaporation, the last one based on ERA5) was performed (Additional file 1: Table S1 and Additional file 3. Table S2A).

### RNA-seq and small RNA-seq experiments

#### Fly strains

To choose which strains to perform RNA-seq and small RNA-seq experiments on, we repeated the desiccation survival experiments with five of the most sensitive and five of the most tolerant strains according to the LT_50_ distribution (Additional file 2: Fig. S1). Three to 4 replicates (except for one strain with 2 replicates) of 14–20 flies each (except for 3 replicates with 8–12 flies) for desiccation conditions and 3–4 replicates (except for 4 strains with 1–2 replicates) of 10–20 flies (except 3 replicas with 8–9 flies) were performed. We confirmed that tolerant strains had a higher LT_50_ compared with sensitive strains. Although the LT_50_ range was different between the two experiments (12 to 30 h vs 16 to 31 h), this was consistent with differences in humidity between the two experiments (13.65% vs 19.47%) (Additional file 3: Table S2A-B, and Additional file 2: Fig. S1). Three tolerant, GIM-012, GIM-024, and COR-023, and three sensitive TOM-08, LUN-07, and MUN-013 strains were chosen for RNA- and small RNA-sequencing (Additional file 3: Table S2C-D). The strains chosen had a high degree of inbreeding and a low degree of variation among biological replicates in the LT_50_ assays (Additional file 1: Table S1 and Additional file 3: Table S2).

#### RNA extraction for qRT-PCR and RNA sequencing

RNA was extracted using *GenElute™* Mammalian Total RNA Miniprep Kit (Sigma-Aldrich) from 30 whole female flies, 4 to 6 days old, per replicate (three replicates per strain analyzed). RNA samples were treated with DNAse I (Thermo Fisher Scientific) following manufacturer’s instructions. RNA concentration was measured using *NanoDrop* spectrophotometer (NanoDrop Technologies) and quality was assessed with Bioanalyzer. Library preparation for RNA sequencing was performed using the Truseq Stranded mRNA Sample Prep kit from Illumina following the manufacturer's instructions. Libraries were sequenced using Illumina 125 bp paired-end reads.

#### RNA extraction for small RNA sequencing

Total RNA was isolated using Trizol (Thermo Fisher Scientific) from three replicates of 30 4–7 day-old female flies after desiccation stress and under control conditions. RNA samples were treated with DNAse I (Thermo Fisher Scientific) following manufacturer’s instructions. Small RNAs were obtained by gel size selection from total RNA using 15% Mini-Protean TBE-Urea Gel (Bio-Rad, #4,566,056). The gel was run at 300 V for 50 min. Fragments between 17 and 30 nucleotides were carefully cut from the gel and were put in an Eppendorf with 250 μL NaCl 0.5 M and then were placed at 4 °C in a rotating wheel o/n. Next, the sample was transferred to Corning Costar Spin-X centrifuge tubes (Merck, CLS8162), spinned and cleaned with standard ethanol washes, and eluted in DEPEC-H2O. Quality check was performed using Bioanalyzer small RNA kit (Agilent). Libraries were sequenced using Illumina Next-Seq 50 bp single-end reads.

#### RT-qPCR

We checked whether the desiccation stress triggered molecular changes in the flies and confirmed that the replicates gave similar results by measuring the expression of *frost* gene which has been reported to be upregulated in desiccation conditions [43]. Primers used were: *frost* forward (5′-CGATTCTTCAGCGGTCTAGG-3′) and *fros*t reverse (5′-CTCGGAAAC GCCAAATTTTA-3′). RT-qPCR data were normalized with *Actin 5* (*Act5*) expression and mRNA abundance of the *frost* gene was compared to that in control samples using the 2(-Delta Delta C(T)) method and one-tailed Student’s *t*-test [106] (Additional file 14: Table S13).

### Data analysis

#### RNA-seq data

Overall, we obtained 22.4 to 42 M (median 31 M) paired-end reads per sample (36 samples in total: three tolerant, three sensitive strains in treated and control conditions and three replicates per strain and condition). Fastq sequence quality was first assessed using *F*ast*QC* (v.0.11.8) (www.bioinformatics.babraham.ac.uk/projects/fastqc) [107]. Adapter and quality trimming was performed using *Cutadapt* (v. 1.18) [108] with the parameters *–quality-cutoff* 20, *-a* AGATCGGAAGAGC and the *paired-end* option. Trimmed reads were then mapped to the *D. melanogaster* genome r6.15 using *STAR* (v.2.6) [109]. On average, 96.3% of the reads mapped to the reference genome. Technical duplications were explored using *dupRadar* [110]. Overall, we found no bias towards a high number of duplicates at low read counts, so we did not remove duplicates from the alignments. We then used *featureCounts* (v.1.6.2) [111] for counting the number of reads mapping to genes (*reverse-stranded* parameter). Overall, 91.81% of the aligned reads were uniquely assigned to a gene feature. We used *RSeQC* (v.2.6.4) (http://rseqc.sourceforge.net/) [112] for determining junction saturation, and we found all samples saturated the number of splice junctions, meaning that the sequencing depth used in the analysis was sufficient*.* Raw sequencing data and matrix of raw counts per gene have been deposited in NCBI's Gene Expression Omnibus [113] and are accessible through GEO Series accession number GSE153850 [73].

#### Transcriptogramer analysis

*Transcriptogramer* R package (v. 1.4.1) was used to perform topological analysis, differential expression (DE), and gene ontology (GO) enrichment analyses [74]. *Transcriptogramer* identifies expression profiles and analyzes GO enrichment of entire genetic systems instead of individual genes. We normalized and filtered raw counts of RNA-seq reads using the functions, *fread ()*, *calcNormFactors()*, and *filterByExpr()* (count per million values higher than 0.5) in the *R data.table* (v. 1.12.2) (https://github.com/Rdatatable/data.table/wiki) and the *edgeR package* (v 3.24.3) [114, 115]. The filtering step was performed to remove those genes that were lowly expressed and, thus, would not be retained in the posterior statistical analysis. Then, we analyzed the processed data using the *Transcriptogramer* pipeline to identify the differential expression of functionally associated genes (hereafter clusters). The workflow of *Transcriptogramer* requires (i) an edge list with the gene connections, which was downloaded from *STRINGdb* (v11.0) with a combined score greater or equal to 800; (ii) an ordered gene list, where genes are sorted by the probability of their products to interact with each other, which was obtained using the *Transcriptogramer* (v 1.0) for Windows (https://lief.if.ufrgs.br/pub/biosoftwares/transcriptogramer/); (iii) expression data, which in this case were the processed reads (described above) of our RNA-seq analysis; and (iv) a dictionary, for mapping proteins to gene identifiers used as expression data row-names. The name of the genes in our data were converted to *Ensembl* Peptide IDs using the *biomaRt R/Bioconductor* package (v 2.38.0) [116] to build a dictionary to map the *Ensembl* Peptide IDs to *Ensembl* Gene IDs. First, the program assigns expression values (obtained from the RNA-seq experiment) to each respective gene in the ordered gene list. Then, the average expression of neighboring genes gets assigned to each gene in the ordered gene list. In order to measure the average expression of functionally associated genes, represented by neighbor genes in the ordered gene list, we must define a sliding window centered on a given gene with a fixed radius. We initially specified three different radii (50, 80, and 125) and finally choose 125, because this gave us the highest number of statistically significant windows per cluster [74]. The *p*-value threshold for FDR correction for the differential expression was set to 0.01.

We then checked if the clusters of genes that were differentially expressed were enriched for specific GO terms representing specific pathways. The p-value threshold for FDR correction for the GO analysis was set to 0.005, and we focused on the first 10 GO terms with the highest adjusted *p*-value for each cluster when interpreting the results.

We run *Transcriptogramer* with (i) the six strains comparing treated and control conditions (“All DEGs”), (ii) the three tolerant strains comparing treated and control conditions (“Tolerant DEGs”), (iii) the three sensitive strains comparing treated and control conditions (“Sensitive DEGs”), and (iv) the six strains comparing tolerant and sensitive strains in basal conditions (“Basal DEGs”).

#### Protein–protein interaction (PPI) network analysis

To identify the differentially expressed hub genes, which are likely to have a greater biological impact as they show a greater number of gene interactions, we did PPI network analysis and estimated the parameters summarizing several network properties [117]. Analysis was performed using *STRING* (v 11), on the previously mentioned four groups (“All DEGs,” “Tolerant DEGs,” “Sensitive DEGs,” “Basal DEGs”). We only considered the results with a minimum required interaction score of 0.8, since those are considered as strongly correlated [118]. We used *experiments* and *co-expression* data as interaction sources. The hub genes were determined using the *Cytoscape* (v.3.7.1) plugin *cytoHubba*, which calculates 11 properties of PPI networks. Among these 11 properties, maximal clique centrality (MCC) is considered one of the most efficient parameters to identify hub genes [78]. We ranked the genes by MCC and considered as hub genes and candidates for being involved in desiccation stress response the 30% of the genes with the highest MCC values.

#### Small RNA-sequencing data

We obtained 52–83 million single-end reads per sample (36 samples in total: three tolerant, three sensitive strains in treated and control conditions and in three biological replicates). Fastq sequence quality was assessed using FastQC (v.0.11.9) [107] and the ones that passed the filters were then used for further analysis. Afterwards, the high-quality Illumina sequencing reads were filtered using BBduk (parameters: *minlen* = *15 qtrim* = *rl ktrim* = *r k* = *21 hdist* = *2 mink* = *8*; http://jgi.doe.gov/data-and-tools/bb-tools/) to remove adapters and ribosomal RNA sequences. The trimmed reads were then analyzed using SPORTS (v.1.0.) [119]: an annotation pipeline designed to optimize the annotation and quantification of canonical and non-canonical small RNAs including tRNA-derived fragments (tRFs) in small RNA sequencing data (https://github.com/junchaoshi/sports1.0). SPORTS (v.1.0.) sequentially maps the cleaned reads against the *D. melanogaster* reference genome (v.6.15), miRBase [120], rRNA database (collected from NCBI), GtRNAdb [121], and piRNA database [122]. On average 82% of the small RNA reads mapped to the *D. melanogaster* reference genome (r6.15). In this study, we focused on the reads mapping to the GtRNAdb (mature tRNAs) database. SPORTS (v.1.0.) was also used to identify the locations of tRFs regarding whether they were derived from 5′ terminus, 3′ terminus, or 3′CCA end of the tRNAs.

Next, to identify the genes that could be targeted by the tRFs, we mapped the tRF sequences—taken from the SPORTS output file—against the full transcripts of the D*. melanogaster* r6.15 transcriptome using RNAhybrid [123] with the following parameters: *(-u 1, -m 18,500, -b 1, -v 1*). Only the first 12nt of each sequence—containing the seed region—were used as query sequences [52]. As Luo et al. (2018) suggested that 5′ fragments might have different properties to 3′ or CCA fragments under stress, all three types of tsRNA fragment were analyzed separately. However, since most of the tRFs were derived from 5′ terminus (63–88%), we focused on those sequences. While two minimum free energy (MFE) cut off points were explored (MFE -20 and -30), we chose the more stringent method. This MFE -30 cut off was also chosen by Seong et al. (2019) [124]. Only transcripts with at least 10 tRF reads mapped in each one of the three replicates per sample were used for further analysis.

Small RNA data is available through GEO Series accession number GSE196669 [83].

### Differentially expressed gene location analysis

Using the *Drosophila Gene Expression Tool* (DGET), we checked the previously reported location and the level of expression of the DEGs in this study [77]. DGET uses modENCODE and RNA-seq experiment data and offers information in several life stages and tissues of *D. melanogaster*. Since we were working with 4–6 day-old females, we only used data from adult, mated 4-day old females. Expression data was available for head, digestive system, carcass, and ovary. We considered the genes with at least high expression (RPKM > 51) based on the DGET database. The enrichment of DEGs in tissues was checked using a hypergeometric test, and the significant *p*-value after a Bonferroni correction was 0.001.

### Gene disruption and RNAi knockdown strains

Three of the hub genes among the ones with the highest MCC values were selected for experimental validation (*nclb*,* Nsun2*, and *Dbp73D*). To determine if the candidate hub genes influence desiccation tolerance, we performed functional validation using a combination of gene disruption and RNAi transgenic lines (Additional file 8: Table S7). The effect of each gene was tested in two different backgrounds, when stocks were available. In the case of *nclb* gene*,* the flies generated with the ubiquitous GAL4 driver were not viable, so we crossed the RNAi lines with the 6g1HR-GAL4-6c (Hikone) driver line, which only affects the expression of the gene of interest in the midgut, Malphigian tubules and fat body, where the *nclb* gene is mostly expressed [125] (Additional file 8: Table S7). To generate the *Nsun2* knockdowns, we crossed strains carrying the RNAi controlled by an *UAS* promoter with flies carrying a ubiquitous GAL4 driver to silence the gene (Additional file 8: Table S7). In the case of the *Dbp73D* gene, to overcome the lethality of the pupae caused by the ubiquitous GAL4 driver, we used an Act5c-GAL4 strain regulated by the temperature sensitive repressor GAL80 (P tubP-GAL80ts), which allowed us to time the activation of the driver. For these crosses, we transferred flies from 25 °C to 29 °C before emerging, to activate the driver that causes the mutation. Since not all offspring of each previously mentioned cross would have inherited the UAS-RNAi construct, we separated the flies with the construct from the ones without in the F1 generation based on the phenotypic markers. In all cases, we did reciprocal crosses of the transgenic lines and the driver strains and all experiments were carried out in the F1 generation. As controls in the experiment, reciprocal crosses with the wild-type strain of each RNAi line and the corresponding drivers were generated (Additional file 8: Table S7).

Besides the four RNAi lines, we analyzed gene disruption strains generated with a P-element transposable element insertion for *nclb* and *Nsun2*. We used the background strain in which the mutant was generated as a control in the experiment (Additional file 8: Table S7).

We checked if the expression of the genes was different in RNAi and gene-disruption strains compared to wild-type strains by performing RT-qPCR analysis (Additional file 8: Table S7). For *Dbp73D*, when we crossed the #108310 female with the P tubP-GAL80ts driver male, we found no difference in the expression in one of the reciprocal crosses and slightly higher expression in the other reciprocal cross (Additional file 8: Table S7). Thus, no further experiments were performed with this RNAi line.

All the desiccation experiments were done with 4–7 day-old females, and at least three replicates per strain were performed, using 6–13 flies/replicate (Additional file 8: Table S7). We followed the desiccation phenotyping protocol described above. Survival curves were analyzed with log-rank test using SPSS statistical software (v.26). We found differences in the survival of the strains in control conditions in only one case. One of the reciprocal crosses for *Dbp73D* (RNAi #36,131 F x Gal80ts M) showed mortality under control conditions (Additional file 8: Table S7). Still, we found that the mortality of these flies under desiccation conditions was higher than under control conditions (long rank test p-value: < 0.0001; Additional file 8: Table S7).

### Analysis of transposable element insertions

We analyzed the TEs previously annotated in our laboratory in three tolerant (GIM-024, GIM-012, COR-023) and three sensitive (LUN-07, TOM-08, MUN-013) strains [81, 82]. Genomes of these strains were sequenced using Oxford Nanopore Technologies and TEs were de novo annotated using REPET package (v.2.5) [126–128]. We did not consider TEs smaller than 120 bp as they are known to have high false positives/negatives rates [82].

We analyzed the TE presence/absence nearby the DEGs in the “All DEGs” and “Basal DEGs” in the six genomes, while nearby “Tolerant DEGs” were analyzed in the three tolerant strains, and “Sensitive DEGs” were analyzed in the three sensitive strains. We focused on the TEs located either inside genes or within 1 kb of distance to the closest gene. We used bedtools closest (v2.29.2); [129] to define TE insertions within 1 kb of each gene (parameters: -k 10, -D ref) using the TE annotations available in the comparative genome browser DrosOmics [130]. To determine the distribution of the DEGs groups nearby TEs, we used chi-square analysis. To perform post hoc analysis, we used the function chisq.posthoc.test() from the R package chisq.posthoc.test (v.0.1.2). The critical *Z* value is − 2.497705, and the *p*-value after Bonferroni correction is 0.0125.

### Desiccation-related phenotypic experiments

In the experiments detailed below, 10 tolerant and 10 sensitive strains were used from the tails of the LT_50_ phenotypic distribution, except for the respiration rate measurements, which were performed with a subset of three tolerant and three sensitive strains (Additional file 2: Fig. S1). In all the experiments, we used 4–7 day-old female flies.

#### Initial water content

The initial water content measurements were performed as described in Gibbs and Matzkin (2001) with some modifications [22]. Briefly, 10 replicates of 10 females from each strain were anesthetized with CO_2_ and placed into microcentrifuge tubes, put at − 80 °C for a few seconds, and then had their body weight measured. The tubes were placed at 55 °C for 72 h, and their dry body weight was measured again. The initial water content was estimated as the difference between wet and dry body mass [28]. To test if the data followed a normal distribution, we used the Shapiro–Wilk normality test. Since the data was not normally distributed (Shapiro-Wilk test *p*-value=5.279e-10), we used Wilcoxon-signed rank test to identify the differences between the two groups.

#### Water loss analysis

Four to five replicates per strain (except one strain with 3 and one strain with 2 replicates) of 5 flies each were anesthetized on ice and their weight was measured. Flies were then transferred to vials containing 3 g of silica (Merck) for 6 h. After this time, their weight was measured again. The silica reduces the humidity to < 20% in about 3 h, so the flies were exposed to low humidity conditions (< 20%) for 3 h. Water loss was calculated as the difference between the initial and final weight after desiccation stress. To identify if the data followed a normal distribution, we used the Shapiro–Wilk normality test. Since the data was not normally distributed (Shapiro-Wilk test *p*-value=0.007856), we used Wilcoxon-signed rank test to identify the differences between the two groups. All the initial water content and water loss measurements were done in a *Mettler Toledo AJ100* microbalance (000.1-g accuracy).

#### Respiration rate measurements

Insect CO_2_ exchange rate was measured with a portable photosynthesis system (Li- 6400XT, Li-Cor Biosciences, Lincoln, Nebraska, USA). We used a 7.5-cm diameter clear conifer chamber (LI-6400–05; 220 cm^3^ approx. volume) to measure the respiration rate (μg CO_2_ g Insect^−1^ min^−1^). The system was calibrated daily for zero water and CO_2_ concentrations; moreover, infrared gas analyzers (IRGAs) were matched before introducing the insects in the chamber (as suggested by 6400–89 Insect Respiration Chamber Manual). Groups of five females, three replicates per strain, were placed in a net container located inside the measuring chamber. The flow rate of the air was set to 150 mol s^−1^, whereas the CO_2_ concentration inside the chamber was fixed to 400 ppm, which is the same concentration found in nature. Measurements were collected every 60 s. We measured for 3 h in normal humidity conditions (65 ± 5%), and then, we reduced the humidity to 20 ± 5% and measured for 3 more hours. In both conditions, only the measurements from the second hour were used for statistical analysis, because flies need at least 1 h to stabilize the respiration after the introduction in the chamber [32], and once in the chamber the rates did not change between the second and the third hour. The chamber was covered with a 2 × 2 cm of dark paper to keep the flies in a less active state. To test if there are differences in the respiration rate of sensitive and tolerant strains, we transformed the respiration rate values using a Yeo-Johnson transformation (bestNormalize v.1.8.2 in R). We then run two GLMM models, a model without interactions (glmm1 = lmer(R_rate_t ~ Condition + Phenotype + (1|Strain/Replicate), res, REML = F)) and a model with interactions (glmm2 = lmer(R_rate_t ~ Condition * Phenotype + (1|Strain/Replicate), res), REML = F), where Condition and Phenotype refers to whether the strains were under control or desiccation conditions and to the sensitive or tolerant phenotype, respectively. We then compared the two models using a likelihood ratio test (LRT).

#### Extraction and analysis of cuticular hydrocarbons

To extract the cuticular hydrocarbons** (**CHCs) 7–10 replicates (except for one strain with 4 replicates) of five flies each per each strain were used. Flies were plunged in 200 μl of *hexane* (> 99% purity, Sigma Aldrich) containing 20 μl of an internal standard (tridecane, 10 ng/μl) and soaked for 9 min. Samples were vortexed gently for one minute, and then the extract was removed and placed in a conical glass insert. The samples were stored at − 20 °C until the final analysis.

Gas chromatography-mass spectrometry analysis was performed using a gas chromatograph (GC Agilent 7890B) coupled with a quadrupole mass spectrometer (MS Agilent 5977A MSD) operating in electron ionization mode (internal ionization source; 70 eV). Two microliters of each sample were injected in the GC injection port held at 260 °C using a split ratio of 1:5. A DB-5 ms fused silica capillary column (30 m × 0.250 mm; film thickness of 0.25 μm) was used for separation using helium as the carrier gas at a constant flow rate of 1.5 mL/min. The temperature program was as follows: 40 °C (1 min), then it was increased with a rate of 5 °C min^−1^ until 110 °C, followed by 10 °C min^−1^ to 300 °C (held 2 min). The mass spectra were recorded from m/z 33 to 450. A C7-C30 n-alkane series (Supelco, Bellefonte, PA) under the same chromatographic conditions was injected in order to calculate the linear retention indices (LRIs). Tentative identification of compounds was based on mass spectra matching the NIST-2014/Wiley 7.0 libraries and comparing the calculated LRIs with those available from the literature [31, 69, 75, 131–133]. The amount (ng/insect) of each component was calculated relative to the internal standard (tridecane, 10 ng/μl). The absolute and relative (%) amount of each component was then calculated.

Descriptive statistical analyses were performed using the R (v.4.0.0.) and SPSS software (v.26). PCA analysis was performed with the log transformed data. Relative % and balanced ratios of desaturated (*D*) and saturated (*S*) compounds were calculated as in Rouault et al. (2004): (*D* − *S*)/(*D* + *S*). Because the data was not normally distributed (Shapiro–Wilk test *p*-value = 9.229 e-11), we used nonparametric tests. The comparison of balanced ratios and amounts of specific compounds were calculated using a Wilcoxon-signed rank test [69, 133]. The correlation between the hydrocarbons and the survival of the flies (LT_100_) was calculated with a Spearman’s correlation test.

### Statistical analysis

All the statistical analyses were performed using R (v.3.5.2) for Mac, unless stated differently [134].

### Supplementary Information

**Additional file 1:** **Table S1.** Inbred strains used in this work and geographical and environmental variables of the populations in which they were collected.

**Additional file 2:** **Figures S1-S2.** **Fig. S1**. Desiccation survival of natural *D. melanogaster *strains. **Fig. S2.** PCA analysis of the CHC of tolerant and sensitive strains.

**Additional file 3:** **Table S2.** Desiccation survival assays including the LT_100_ and LT_50_ values.

**Additional file 4:** **Table S3.** Differentially expressed genes (DEGs) and enriched gene ontology (GO) categories for the clusters of differentially expressed genes (DEGs) identified by Transcriptogramer.

**Additional file 5: Table S4.** Overlap among differentially expressed genes in this study and in previous studies.

**Additional file 6: Table S5.** DGET tool results for each DEG group tested.

**Additional file 7: Table S6**. Results of the PPI network analysis performed using STRING and MCC calculation performed using Cytohubba.

**Additional file 8: Table S7.** Functional validation of candidate desiccation-responsive genes.

**Additional file 9: Table S8.** TEs located nearby DEGs.

**Additional file 10: Table S9**. Analyis of the genes that gain or lose tRFs targeting in response to desiccation stress in tolerant and sensitive strains.

**Additional file 11: Table S10.** Water content and water loss measurements with the 10 most sensitive and 10 most tolerant strains according to the LT_50_ distribution.

**Additional file 12: Table S11.** Respirometry measurements and analysis in tolerant and sensitive strains in control and in desiccation stress conditions.

**Additional file 13: Table S12.** Results of the cuticular hydrocarbon analysis.

**Additional file 14: Table S13.** qRT-PCR results of the *frost *gene for the six strains used in the RNA-seq experiment.

## Data Availability

All data generated or analyzed during this study are included in this published article, its supplementary information files, and publicly available repositories. Raw sequencing RNA-seq data and matrix of raw counts per gene have been deposited in NCBI’s Gene Expression Omnibus (112) and are accessible through GEO Series accession number GSE153850. Raw small RNA sequencing data and processed data files (results of SPORTS and RNAhybrid analysis) are accessible through GEO Series accession number GSE196669.

## Abbreviations

SNP: Single-nucleotide polymorphism
TE: Transposable element
CHC: Cuticular hydrocarbon
tRF: tRNA-derived fragment
LT: Lethal time
DEG: Differentially expressed gene
DGET: Drosophila Gene Expression Tool
MCC: Maximal clique centrality
GLMM: Generalized linear mixed model
LRT: Likelihood ratio test
LRI: Linear retention index
VIF: Variance inflation factor
MFE: Minimum free energy
FDR: False discovery rate
