# Supplementary material for: Gene expression differences consistent with water loss reduction underlie desiccation tolerance of natural Drosophila populations
Source: BMC Biol. 2023 Feb 16;21:35. doi: 10.1186/s12915-023-01530-4 (PMC9933328; doi:10.1186/s12915-023-01530-4)
Supplement: Supplementary file 2 — Additional file 2: Figures S1-S2. Fig. S1. Desiccation survival of natural D. melanogaster strains. Fig. S2. PCA analysis of the CHC of tolerant and sensitive strains. [file 12915_2023_1530_MOESM2_ESM.docx]

**Additional file 2: Figure S1**. **Desiccation survival of natural *D. melanogaster* strains**.
**A)** Bar plot showing the LT_50_ values for each of the strains analysed in this work. Y axis shows the average lethal time 50 (LT_50_) *i.e.* the hour when 50% of the flies in all the replicates are dead. X axis represents the individual strains. The strains marked by a star (black and red) were used for water content, water loss and cuticular hydrocarbon (CHC) analysis. The strains marked with red stars were used to repeat the phenotyping to choose the strains for the transcriptomic analysis. The strains with red circles were used for RNA-sequencing and respirometry analysis. The sensitive strain ES_Gua_15_9 was not involved in further analysis because the stock died. **B)** LT_100_ (left) and LT_50_ (right) values of the desiccation phenotyping experiments with five strains from the extremes of the phenotypic distribution. The Y axes show the hours (LT_100_, LT_50_).

**A)**


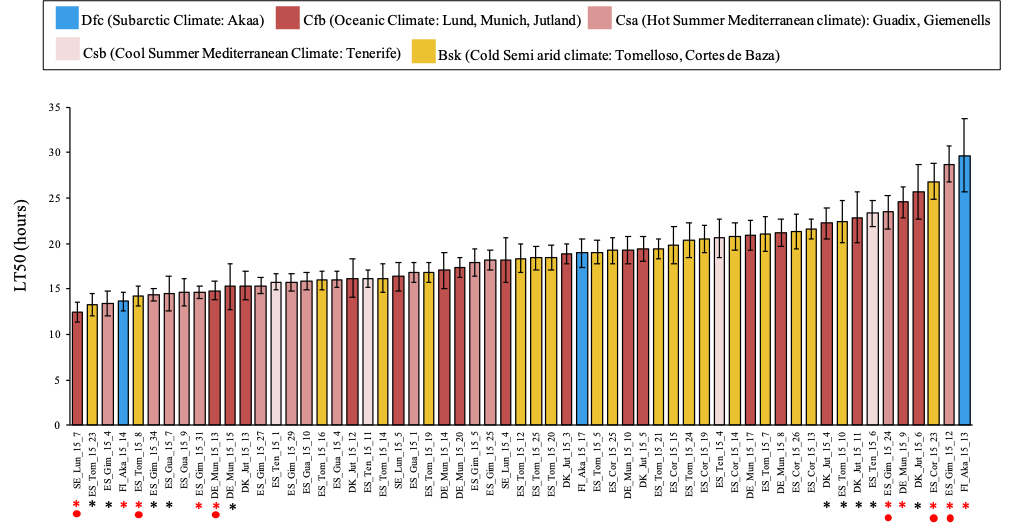


**B)**


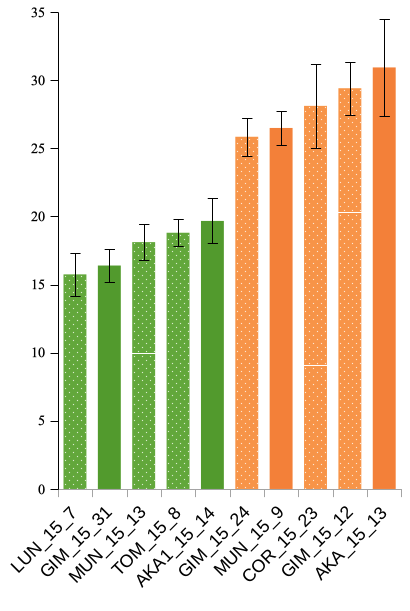


LT50 values (hours)


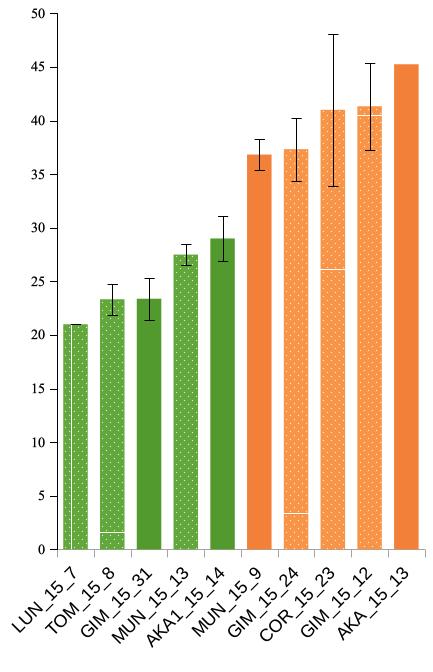


Resistant

Sensitive

LT100 values (hours)

**Figure S2.** PCA analysis of the CHC of tolerant and sensitive strains. **A)** PC1 and PC2 of the CHC variability of the 20 strains from the phenotypic extremes: 10 most tolerant and 10 most sensitive strains **B)** PC1 and PC2 of the CHC variability for the 6 strains used for RNA-sequencing.

**A)**

**B)**
